# 3-Methyl-*N*-phen­ylbenzamide

**DOI:** 10.1107/S1600536808008143

**Published:** 2008-03-29

**Authors:** B. Thimme Gowda, Sabine Foro, B. P. Sowmya, Hartmut Fuess

**Affiliations:** a Department of Chemistry, Mangalore University, Mangalagangotri 574 199, Mangalore, India; b Institute of Materials Science, Darmstadt University of Technology, Petersenstrasse 23, Darmstadt, D-64287, Germany

## Abstract

The conformation of the C=O bond in the structure of the title compound, C_14_H_13_NO, is *anti* to the *meta*-methyl substituent in the benzoyl ring. The conformations of the N—H and C=O bonds in the amide group are also *anti* to each other. The asymmetric unit of the structure contains two mol­ecules. The bond parameters are similar to those in *N*-(phen­yl)benzamide, 2-methyl-*N*-(phenyl)­benz­amide and other benzanilides. The amide group –NHCO– forms dihedral angles of 20.97 (34) and 45.65 (19)° with the benzoyl rings, and 41.54 (25) and 31.87 (29)° with the aniline rings, in the two independent mol­ecules. The benzoyl and aniline rings adopt dihedral angles of 22.17 (18) and 75.86 (12)° in the two independent mol­ecules. In the crystal structure, mol­ecules are linked into chains by inter­molecular N—H⋯O hydrogen bonds.

## Related literature

For related literature, see: Gowda *et al.* (2003 ▶, 2008*a* ▶,*b* ▶).
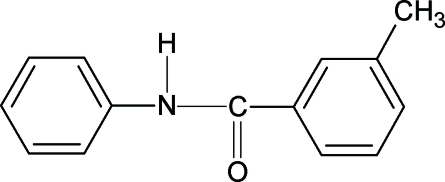


## Experimental

### 

#### Crystal data

C_14_H_13_NO
*M* *_r_* = 211.25
Monoclinic, 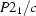

*a* = 16.947 (2) Å
*b* = 15.531 (1) Å
*c* = 8.623 (1) Å
β = 93.35 (1)°
*V* = 2265.7 (4) Å^3^
*Z* = 8
Cu *K*α radiation
μ = 0.62 mm^−1^
*T* = 299 (2) K
0.60 × 0.10 × 0.05 mm

#### Data collection

Enraf–Nonius CAD4 diffractometer
Absorption correction: none
4339 measured reflections
4039 independent reflections
2466 reflections with *I* > 2σ(*I*)
*R*_int_ = 0.034
3 standard reflections frequency: 120 min intensity decay: 1.5%

#### Refinement

*R*[*F* ^2^ > 2σ(*F* ^2^)] = 0.082
*wR*(*F* ^2^) = 0.240
*S* = 1.03
4039 reflections
291 parameters
H-atom parameters constrained
Δρ_max_ = 0.35 e Å^−3^
Δρ_min_ = −0.39 e Å^−3^

### 

Data collection: *CAD-4-PC Software* (Enraf–Nonius, 1996 ▶); cell refinement: *CAD-4-PC Software*; data reduction: *REDU4* (Stoe & Cie, 1987 ▶); program(s) used to solve structure: *SHELXS97* (Sheldrick, 2008 ▶); program(s) used to refine structure: *SHELXL97* (Sheldrick, 2008 ▶); molecular graphics: *PLATON* (Spek, 2003 ▶); software used to prepare material for publication: *SHELXL97*.

## Supplementary Material

Crystal structure: contains datablocks I, global. DOI: 10.1107/S1600536808008143/dn2328sup1.cif

Structure factors: contains datablocks I. DOI: 10.1107/S1600536808008143/dn2328Isup2.hkl

Additional supplementary materials:  crystallographic information; 3D view; checkCIF report

## Figures and Tables

**Table 1:** Hydrogen-bond geometry (Å, °)

*D*—H⋯*A*	*D*—H	H⋯*A*	*D*⋯*A*	*D*—H⋯*A*
N1—H1N⋯O1^i^	0.86	2.16	2.968 (4)	157
N2—H2N⋯O2^ii^	0.86	2.01	2.853 (3)	168

Symmetry codes: (i) 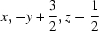; (ii) 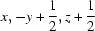.

**Fig. 1.**
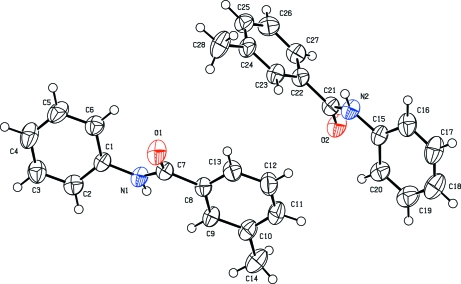
Molecular structure of the title compound, showing the atom labeling scheme. The displacement ellipsoids are drawn at the 50% probability level. H atoms are represented as small spheres of arbitrary radii.

**Fig. 2.**
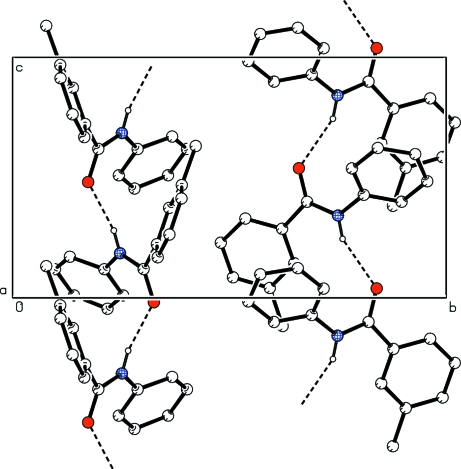
Molecular packing of the title compound with hydrogen bonding shown as dashed lines.

## supplementary crystallographic information

### Comment

As part of a study of the substituent effects on the structures of benzanilides, in the present work, the structure of 3-methyl-*N*-(phenyl)benzamide (NP3MBA) has been determined (Gowda *et al.*, 2003, 2008*a*,*b*).

The asymmetric unit of the structure of NP3MBA contains two molecules (Fig. 1). The conformation of the C═O bonds are anti to the *meta*-methyl substituents in the benzoyl phenyl rings. The conformations of the N—H and C═O bonds in the –NH—CO– groups are also anti to each other. The bond parameters in NP3MBA are similar to those in *N*-(phenyl)benzamide, 2-methyl-*N*-(phenyl)benzamide and other benzanilides (Gowda *et al.*, 2003, 2008*a*,*b*). The amide group –NHCO– forms the dihedral angles of 20.97 (34)° (molecule 1) and 45.65(0.19) (molecule 2) with the benzoyl ring, and 41.54 (25)° (molecule 1), 31.87(0.29) (molecule 2) with the aniline ring. The benzoyl and the aniline rings have the dihedral angles of 22.17 (18)°) (molecule 1) and 75.86(0.12) (molecule 2).

The packing diagram of NP3MBA molecules showing the hydrogen bonds N1—H1N···O1, N2—H2N···O2 (Table 1) involved in the formation of molecular chain is shown in Fig. 2.

### Experimental

The title compound was prepared according to the literature method (Gowda *et al.*, 2003). The purity of the compound was checked by determining its melting point. It was characterized by recording its infrared and NMR spectra. Single crystals of the title compound were obtained from an ethanolic solution and used for X-ray diffraction studies at room temperature.

### Refinement

The NH atom was located in difference map with N—H = 0.86 Å. The other H atoms were positioned with idealized geometry using a riding model with C—H = 0.93–0.96 Å. All H atoms were refined with isotropic displacement parameters (set to 1.2 times of the *U*_eq_ of the parent atom).

### Crystal data

**Table:**

C_14_H_13_NO	*F*_000_ = 896
*M**_r_* = 211.25	*D*_x_ = 1.239 Mg m^−^^3^
Monoclinic, *P*2_1_/*c*	Cu *K*α radiation λ = 1.54180 Å
Hall symbol: -P 2ybc	Cell parameters from 25 reflections
*a* = 16.947 (2) Å	θ = 5.7–21.0º
*b* = 15.531 (1) Å	µ = 0.62 mm^−^^1^
*c* = 8.623 (1) Å	*T* = 299 (2) K
β = 93.35 (1)º	Long needle, colourless
*V* = 2265.7 (4) Å^3^	0.60 × 0.10 × 0.05 mm
*Z* = 8	

### Data collection

**Table:**

Enraf–Nonius CAD4 diffractometer	*R*_int_ = 0.034
Radiation source: fine-focus sealed tube	θ_max_ = 66.9º
Monochromator: graphite	θ_min_ = 2.6º
*T* = 299(2) K	*h* = −20→20
ω/2θ scans	*k* = −1→18
Absorption correction: none	*l* = −10→0
4339 measured reflections	3 standard reflections
4039 independent reflections	every 120 min
2466 reflections with *I* > 2σ(*I*)	intensity decay: 1.5%

### Refinement

**Table:**

Refinement on *F*^2^	Secondary atom site location: difference Fourier map
Least-squares matrix: full	Hydrogen site location: inferred from neighbouring sites
*R*[*F*^2^ > 2σ(*F*^2^)] = 0.082	H-atom parameters constrained
*wR*(*F*^2^) = 0.241	*w* = 1/[σ^2^(*F*_o_^2^) + (0.153*P*)^2^] where *P* = (*F*_o_^2^ + 2*F*_c_^2^)/3
*S* = 1.03	(Δ/σ)_max_ = 0.003
4039 reflections	Δρ_max_ = 0.36 e Å^−^^3^
291 parameters	Δρ_min_ = −0.39 e Å^−^^3^
Primary atom site location: structure-invariant direct methods	Extinction correction: none

### Special details

**Table:**

Geometry. All e.s.d.'s (except the e.s.d. in the dihedral angle between two l.s. planes) are estimated using the full covariance matrix. The cell e.s.d.'s are taken into account individually in the estimation of e.s.d.'s in distances, angles and torsion angles; correlations between e.s.d.'s in cell parameters are only used when they are defined by crystal symmetry. An approximate (isotropic) treatment of cell e.s.d.'s is used for estimating e.s.d.'s involving l.s. planes.
Refinement. Refinement of *F*^2^ against ALL reflections. The weighted *R*-factor *wR* and goodness of fit *S* are based on *F*^2^, conventional *R*-factors *R* are based on *F*, with *F* set to zero for negative *F*^2^. The threshold expression of *F*^2^ > σ(*F*^2^) is used only for calculating *R*-factors(gt) *etc*. and is not relevant to the choice of reflections for refinement. *R*-factors based on *F*^2^ are statistically about twice as large as those based on *F*, and *R*- factors based on ALL data will be even larger.

### Fractional atomic coordinates and isotropic or equivalent isotropic displacement parameters (Å^2^)

**Table:**

	*x*	*y*	*z*	*U*_iso_*/*U*_eq_	
O1	0.24023 (18)	0.66029 (17)	0.5392 (3)	0.0558 (8)	
N1	0.26569 (16)	0.75059 (19)	0.3424 (3)	0.0399 (7)	
H1N	0.2562	0.7611	0.2452	0.048*	
C1	0.3146 (2)	0.8100 (2)	0.4286 (3)	0.0363 (7)	
C2	0.3052 (2)	0.8972 (2)	0.3996 (4)	0.0457 (9)	
H2	0.2686	0.9159	0.3224	0.055*	
C3	0.3502 (3)	0.9564 (3)	0.4849 (5)	0.0559 (10)	
H3	0.3435	1.0150	0.4660	0.067*	
C4	0.4049 (3)	0.9287 (3)	0.5978 (5)	0.0662 (12)	
H4	0.4342	0.9687	0.6571	0.079*	
C5	0.4163 (3)	0.8422 (3)	0.6235 (5)	0.0611 (12)	
H5	0.4545	0.8237	0.6980	0.073*	
C6	0.3711 (2)	0.7827 (3)	0.5385 (4)	0.0481 (9)	
H6	0.3789	0.7241	0.5557	0.058*	
C7	0.2328 (2)	0.6791 (2)	0.3997 (4)	0.0378 (8)	
C8	0.1875 (2)	0.6216 (2)	0.2875 (4)	0.0382 (8)	
C9	0.1563 (2)	0.6497 (2)	0.1438 (4)	0.0395 (8)	
H9	0.1620	0.7071	0.1158	0.047*	
C10	0.1167 (2)	0.5932 (3)	0.0411 (4)	0.0469 (9)	
C11	0.1086 (2)	0.5083 (3)	0.0854 (5)	0.0553 (10)	
H11	0.0830	0.4695	0.0173	0.066*	
C12	0.1378 (3)	0.4802 (3)	0.2293 (5)	0.0598 (11)	
H12	0.1308	0.4232	0.2587	0.072*	
C13	0.1775 (2)	0.5369 (2)	0.3297 (5)	0.0505 (9)	
H13	0.1976	0.5177	0.4263	0.061*	
C14	0.0866 (3)	0.6228 (3)	−0.1174 (4)	0.0639 (12)	
H14A	0.0874	0.6846	−0.1213	0.077*	
H14B	0.0334	0.6027	−0.1380	0.077*	
H14C	0.1197	0.6001	−0.1942	0.077*	
O2	0.26730 (16)	0.32575 (17)	−0.0183 (3)	0.0498 (7)	
N2	0.22500 (17)	0.24790 (19)	0.1854 (3)	0.0416 (7)	
H2N	0.2375	0.2335	0.2799	0.050*	
C15	0.1546 (2)	0.2116 (2)	0.1191 (4)	0.0396 (8)	
C16	0.1310 (3)	0.1321 (2)	0.1709 (4)	0.0538 (10)	
H16	0.1638	0.1014	0.2413	0.065*	
C17	0.0593 (3)	0.0978 (3)	0.1193 (5)	0.0673 (13)	
H17	0.0429	0.0453	0.1581	0.081*	
C18	0.0117 (3)	0.1414 (3)	0.0100 (5)	0.0664 (12)	
H18	−0.0365	0.1181	−0.0258	0.080*	
C19	0.0360 (3)	0.2189 (3)	−0.0454 (5)	0.0603 (11)	
H19	0.0046	0.2477	−0.1207	0.072*	
C20	0.1070 (2)	0.2549 (3)	0.0098 (4)	0.0453 (9)	
H20	0.1226	0.3083	−0.0268	0.054*	
C21	0.2752 (2)	0.3022 (2)	0.1197 (4)	0.0386 (8)	
C22	0.3426 (2)	0.3328 (2)	0.2230 (4)	0.0384 (8)	
C23	0.3317 (2)	0.3609 (2)	0.3741 (4)	0.0427 (8)	
H23	0.2813	0.3599	0.4114	0.051*	
C24	0.3942 (2)	0.3902 (2)	0.4689 (4)	0.0458 (9)	
C25	0.4691 (2)	0.3895 (2)	0.4135 (5)	0.0503 (9)	
H25	0.5121	0.4079	0.4772	0.060*	
C26	0.4808 (2)	0.3617 (3)	0.2633 (5)	0.0529 (10)	
H26	0.5314	0.3614	0.2270	0.063*	
C27	0.4178 (2)	0.3347 (2)	0.1686 (4)	0.0462 (9)	
H27	0.4257	0.3176	0.0673	0.055*	
C28	0.3808 (3)	0.4223 (3)	0.6316 (5)	0.0706 (14)	
H28A	0.3665	0.3748	0.6955	0.085*	
H28B	0.3391	0.4642	0.6270	0.085*	
H28C	0.4285	0.4483	0.6753	0.085*	

### Atomic displacement parameters (Å^2^)

**Table:**

	*U*^11^	*U*^22^	*U*^33^	*U*^12^	*U*^13^	*U*^23^
O1	0.095 (2)	0.0574 (16)	0.0143 (12)	−0.0142 (14)	−0.0011 (12)	0.0030 (10)
N1	0.0552 (17)	0.0488 (16)	0.0154 (12)	−0.0071 (14)	−0.0001 (12)	−0.0003 (11)
C1	0.0451 (18)	0.0481 (19)	0.0160 (14)	−0.0036 (15)	0.0039 (13)	−0.0036 (13)
C2	0.056 (2)	0.054 (2)	0.0275 (18)	−0.0009 (17)	0.0043 (16)	0.0017 (15)
C3	0.076 (3)	0.047 (2)	0.044 (2)	−0.0084 (19)	0.001 (2)	−0.0009 (17)
C4	0.087 (3)	0.065 (3)	0.045 (2)	−0.020 (2)	−0.006 (2)	−0.011 (2)
C5	0.068 (3)	0.073 (3)	0.040 (2)	−0.011 (2)	−0.016 (2)	0.002 (2)
C6	0.056 (2)	0.051 (2)	0.037 (2)	−0.0034 (17)	−0.0014 (17)	0.0017 (16)
C7	0.0488 (19)	0.0458 (19)	0.0191 (15)	0.0027 (15)	0.0043 (14)	−0.0037 (14)
C8	0.048 (2)	0.0444 (19)	0.0229 (16)	−0.0005 (15)	0.0054 (14)	−0.0051 (13)
C9	0.0483 (19)	0.0498 (19)	0.0204 (16)	−0.0092 (16)	0.0036 (14)	−0.0035 (14)
C10	0.050 (2)	0.061 (2)	0.0303 (19)	−0.0054 (17)	0.0034 (16)	−0.0093 (16)
C11	0.059 (2)	0.059 (2)	0.047 (2)	−0.011 (2)	0.0019 (19)	−0.0190 (19)
C12	0.079 (3)	0.042 (2)	0.058 (3)	−0.008 (2)	−0.005 (2)	−0.0042 (18)
C13	0.069 (3)	0.045 (2)	0.037 (2)	−0.0001 (18)	−0.0004 (18)	0.0015 (16)
C14	0.069 (3)	0.090 (3)	0.031 (2)	−0.014 (2)	−0.0042 (19)	−0.008 (2)
O2	0.0733 (18)	0.0608 (16)	0.0150 (11)	−0.0113 (13)	0.0008 (11)	0.0021 (10)
N2	0.0548 (17)	0.0534 (17)	0.0163 (13)	−0.0067 (14)	−0.0002 (12)	0.0036 (12)
C15	0.050 (2)	0.050 (2)	0.0192 (15)	−0.0040 (16)	0.0036 (14)	−0.0074 (14)
C16	0.079 (3)	0.052 (2)	0.0304 (19)	−0.008 (2)	0.0043 (18)	−0.0029 (16)
C17	0.088 (3)	0.070 (3)	0.044 (2)	−0.029 (3)	0.006 (2)	−0.008 (2)
C18	0.066 (3)	0.089 (3)	0.044 (2)	−0.021 (2)	0.003 (2)	−0.014 (2)
C19	0.062 (2)	0.081 (3)	0.037 (2)	0.004 (2)	−0.0015 (19)	−0.008 (2)
C20	0.052 (2)	0.058 (2)	0.0252 (17)	0.0003 (18)	0.0017 (15)	−0.0019 (15)
C21	0.0509 (19)	0.0455 (19)	0.0197 (15)	0.0023 (15)	0.0041 (14)	−0.0045 (13)
C22	0.050 (2)	0.0424 (19)	0.0223 (16)	−0.0011 (15)	0.0013 (14)	0.0011 (13)
C23	0.052 (2)	0.051 (2)	0.0248 (17)	−0.0048 (16)	0.0032 (15)	−0.0019 (15)
C24	0.060 (2)	0.048 (2)	0.0282 (18)	−0.0052 (17)	−0.0064 (17)	−0.0034 (15)
C25	0.054 (2)	0.053 (2)	0.043 (2)	−0.0016 (17)	−0.0085 (18)	0.0002 (17)
C26	0.047 (2)	0.060 (2)	0.052 (2)	0.0024 (18)	0.0040 (18)	0.0027 (19)
C27	0.054 (2)	0.053 (2)	0.0325 (19)	0.0030 (17)	0.0077 (16)	−0.0011 (16)
C28	0.085 (3)	0.092 (3)	0.034 (2)	−0.021 (3)	−0.002 (2)	−0.023 (2)

### Geometric parameters (Å, °)

**Table:**

O1—C7	1.237 (4)	O2—C21	1.244 (4)
N1—C7	1.348 (4)	N2—C21	1.347 (4)
N1—C1	1.421 (4)	N2—C15	1.409 (4)
N1—H1N	0.8600	N2—H2N	0.8600
C1—C6	1.373 (5)	C15—C20	1.379 (5)
C1—C2	1.385 (5)	C15—C16	1.382 (5)
C2—C3	1.379 (5)	C16—C17	1.376 (6)
C2—H2	0.9300	C16—H16	0.9300
C3—C4	1.373 (6)	C17—C18	1.381 (7)
C3—H3	0.9300	C17—H17	0.9300
C4—C5	1.373 (6)	C18—C19	1.368 (6)
C4—H4	0.9300	C18—H18	0.9300
C5—C6	1.384 (5)	C19—C20	1.386 (5)
C5—H5	0.9300	C19—H19	0.9300
C6—H6	0.9300	C20—H20	0.9300
C7—C8	1.496 (5)	C21—C22	1.484 (5)
C8—C13	1.378 (5)	C22—C27	1.384 (5)
C8—C9	1.389 (5)	C22—C23	1.397 (5)
C9—C10	1.391 (5)	C23—C24	1.376 (5)
C9—H9	0.9300	C23—H23	0.9300
C10—C11	1.382 (6)	C24—C25	1.383 (6)
C10—C14	1.503 (5)	C24—C28	1.519 (5)
C11—C12	1.380 (6)	C25—C26	1.391 (6)
C11—H11	0.9300	C25—H25	0.9300
C12—C13	1.382 (5)	C26—C27	1.372 (5)
C12—H12	0.9300	C26—H26	0.9300
C13—H13	0.9300	C27—H27	0.9300
C14—H14A	0.9600	C28—H28A	0.9600
C14—H14B	0.9600	C28—H28B	0.9600
C14—H14C	0.9600	C28—H28C	0.9600
			
C7—N1—C1	125.7 (3)	C21—N2—C15	128.3 (3)
C7—N1—H1N	117.1	C21—N2—H2N	115.8
C1—N1—H1N	117.1	C15—N2—H2N	115.8
C6—C1—C2	119.6 (3)	C20—C15—C16	119.2 (3)
C6—C1—N1	121.5 (3)	C20—C15—N2	122.0 (3)
C2—C1—N1	118.9 (3)	C16—C15—N2	118.8 (3)
C3—C2—C1	120.2 (4)	C17—C16—C15	120.6 (4)
C3—C2—H2	119.9	C17—C16—H16	119.7
C1—C2—H2	119.9	C15—C16—H16	119.7
C4—C3—C2	119.8 (4)	C16—C17—C18	120.0 (4)
C4—C3—H3	120.1	C16—C17—H17	120.0
C2—C3—H3	120.1	C18—C17—H17	120.0
C5—C4—C3	120.3 (4)	C19—C18—C17	119.6 (4)
C5—C4—H4	119.8	C19—C18—H18	120.2
C3—C4—H4	119.8	C17—C18—H18	120.2
C4—C5—C6	119.9 (4)	C18—C19—C20	120.6 (4)
C4—C5—H5	120.0	C18—C19—H19	119.7
C6—C5—H5	120.0	C20—C19—H19	119.7
C1—C6—C5	120.1 (4)	C15—C20—C19	119.9 (4)
C1—C6—H6	120.0	C15—C20—H20	120.0
C5—C6—H6	120.0	C19—C20—H20	120.0
O1—C7—N1	122.0 (3)	O2—C21—N2	123.4 (3)
O1—C7—C8	120.4 (3)	O2—C21—C22	121.1 (3)
N1—C7—C8	117.5 (3)	N2—C21—C22	115.5 (3)
C13—C8—C9	119.3 (3)	C27—C22—C23	118.9 (3)
C13—C8—C7	117.8 (3)	C27—C22—C21	119.7 (3)
C9—C8—C7	123.0 (3)	C23—C22—C21	121.4 (3)
C8—C9—C10	121.0 (3)	C24—C23—C22	121.2 (3)
C8—C9—H9	119.5	C24—C23—H23	119.4
C10—C9—H9	119.5	C22—C23—H23	119.4
C11—C10—C9	118.5 (4)	C23—C24—C25	118.9 (3)
C11—C10—C14	120.7 (4)	C23—C24—C28	120.4 (4)
C9—C10—C14	120.8 (4)	C25—C24—C28	120.7 (4)
C12—C11—C10	121.0 (4)	C24—C25—C26	120.5 (4)
C12—C11—H11	119.5	C24—C25—H25	119.7
C10—C11—H11	119.5	C26—C25—H25	119.7
C11—C12—C13	119.9 (4)	C27—C26—C25	120.1 (4)
C11—C12—H12	120.1	C27—C26—H26	120.0
C13—C12—H12	120.1	C25—C26—H26	120.0
C8—C13—C12	120.4 (4)	C26—C27—C22	120.4 (4)
C8—C13—H13	119.8	C26—C27—H27	119.8
C12—C13—H13	119.8	C22—C27—H27	119.8
C10—C14—H14A	109.5	C24—C28—H28A	109.5
C10—C14—H14B	109.5	C24—C28—H28B	109.5
H14A—C14—H14B	109.5	H28A—C28—H28B	109.5
C10—C14—H14C	109.5	C24—C28—H28C	109.5
H14A—C14—H14C	109.5	H28A—C28—H28C	109.5
H14B—C14—H14C	109.5	H28B—C28—H28C	109.5
			
C7—N1—C1—C6	40.6 (5)	C21—N2—C15—C20	−32.3 (5)
C7—N1—C1—C2	−139.9 (4)	C21—N2—C15—C16	150.9 (4)
C6—C1—C2—C3	−2.6 (5)	C20—C15—C16—C17	−2.7 (6)
N1—C1—C2—C3	178.0 (3)	N2—C15—C16—C17	174.2 (4)
C1—C2—C3—C4	0.6 (6)	C15—C16—C17—C18	2.7 (7)
C2—C3—C4—C5	1.6 (7)	C16—C17—C18—C19	−0.6 (7)
C3—C4—C5—C6	−1.9 (7)	C17—C18—C19—C20	−1.5 (7)
C2—C1—C6—C5	2.3 (5)	C16—C15—C20—C19	0.7 (5)
N1—C1—C6—C5	−178.2 (4)	N2—C15—C20—C19	−176.2 (3)
C4—C5—C6—C1	−0.1 (6)	C18—C19—C20—C15	1.4 (6)
C1—N1—C7—O1	3.1 (6)	C15—N2—C21—O2	−3.5 (6)
C1—N1—C7—C8	−176.0 (3)	C15—N2—C21—C22	177.0 (3)
O1—C7—C8—C13	−21.2 (5)	O2—C21—C22—C27	−44.0 (5)
N1—C7—C8—C13	157.9 (3)	N2—C21—C22—C27	135.6 (3)
O1—C7—C8—C9	159.5 (3)	O2—C21—C22—C23	135.1 (4)
N1—C7—C8—C9	−21.4 (5)	N2—C21—C22—C23	−45.3 (5)
C13—C8—C9—C10	−1.4 (5)	C27—C22—C23—C24	−0.1 (5)
C7—C8—C9—C10	177.9 (3)	C21—C22—C23—C24	−179.2 (3)
C8—C9—C10—C11	0.4 (6)	C22—C23—C24—C25	−1.5 (6)
C8—C9—C10—C14	−176.9 (4)	C22—C23—C24—C28	178.6 (4)
C9—C10—C11—C12	1.1 (6)	C23—C24—C25—C26	1.5 (6)
C14—C10—C11—C12	178.4 (4)	C28—C24—C25—C26	−178.7 (4)
C10—C11—C12—C13	−1.6 (7)	C24—C25—C26—C27	0.1 (6)
C9—C8—C13—C12	0.9 (6)	C25—C26—C27—C22	−1.7 (6)
C7—C8—C13—C12	−178.5 (4)	C23—C22—C27—C26	1.7 (5)
C11—C12—C13—C8	0.6 (7)	C21—C22—C27—C26	−179.2 (3)

### Hydrogen-bond geometry (Å, °)

**Table:**

*D*—H···*A*	*D*—H	H···*A*	*D*···*A*	*D*—H···*A*
N1—H1N···O1^i^	0.86	2.16	2.968 (4)	157
N2—H2N···O2^ii^	0.86	2.01	2.853 (3)	168

Symmetry codes: (i) *x*, −*y*+3/2, *z*−1/2; (ii) *x*, −*y*+1/2, *z*+1/2.
